# Case report: Spiller syndrome initially mimicking vestibular neuritis

**DOI:** 10.3389/fneur.2022.1072220

**Published:** 2023-01-09

**Authors:** Huiyuan Wang, Tianming Shi, Yafei Shang, Xinyi Chen, Jie Xu, Yu Geng

**Affiliations:** ^1^Department of Clinical Medicine, Bengbu Medical College, Bengbu, China; ^2^Center for Rehabilitation Medicine, Department of Neurology, Zhejiang Provincial People's Hospital (Affiliated People's Hospital, Hangzhou Medical College), Hangzhou, China

**Keywords:** Spiller syndrome, vestibular neuritis (VN), nucleus prepositus hypoglossal, ischemic stroke (IS), vertigo, nystagmus

## Abstract

Spiller syndrome is a rare subtype of medial medullary infarction (MMI). Herein, we report on a patient with progressing stroke who presented with the initial features of acute peripheral vestibulopathy and MMI (Spiller syndrome), as confirmed by magnetic resonance imaging (MRI). A 42-year-old man experienced acute persistent vertigo with nausea, vomiting, and severe gait instability for 6 h before presenting to the emergency department. He exhibited spontaneous right-beating horizontal-torsional nystagmus that intensified on rightward gaze. The patient fell to the left side during the Romberg test. Cranial computed tomography (CT) performed immediately upon admission did not provide evidence for ischemia or hemorrhage of the brainstem and cerebellum; however, the symptoms underwent exacerbation 4 h after admission, manifesting as left-sided limb weakness and dysarthria, without dysphagia. Furthermore, bedside examination revealed difficulty in extending the tongue to the right, positive left Babinski's sign, and abnormal vibration and position sense in the paralyzed limb. Head impulse test recording revealed a normal gain in the vestibulo-ocular reflex, and numerous consistent covert corrective saccades were captured upon turning the head to the left side. Cranial MRI depicted an acute infarct confined to the right side of the medial medulla, which met the diagnostic criteria for Spiller syndrome. Our study underscores the importance of considering the possibility of a nucleus prepositus hypoglossi lesion even if the signs and symptoms support the diagnosis of peripheral lesions in patients with acute vestibular syndrome exhibiting vascular risk factors.

## 1. Introduction

Spiller syndrome, a rare subtype of medial medullary infarction (MMI), is characterized by a triad of contralateral hemiparesis sparing the face, the contralateral loss of deep sensation, and ipsilateral hypoglossal paralysis (1, 2). Owing to the complexity and variability of the vascular supply to the medial medulla oblongata (3, 4), a typical triadic presentation of Spiller syndrome is uncommon (5–7).

Acute vestibular syndrome (AVS) manifests as recent-onset continuous vertigo, nausea, vomiting, motion intolerance, and gait instability lasting from days to weeks (8). Vestibular neuritis (9) is a common etiology of AVS. However, it also occurs in patients with stroke involving the cerebellum or brainstem, i.e., pseudo-vestibular neuritis (10, 11), which has rarely been reported in Spiller syndrome.

This case report describes a patient with progressing stroke who initially presented with the features of both acute peripheral vestibulopathy and MMI (Spiller syndrome), as confirmed by magnetic resonance imaging (MRI).

## 2. Case description

**Figure 1:**
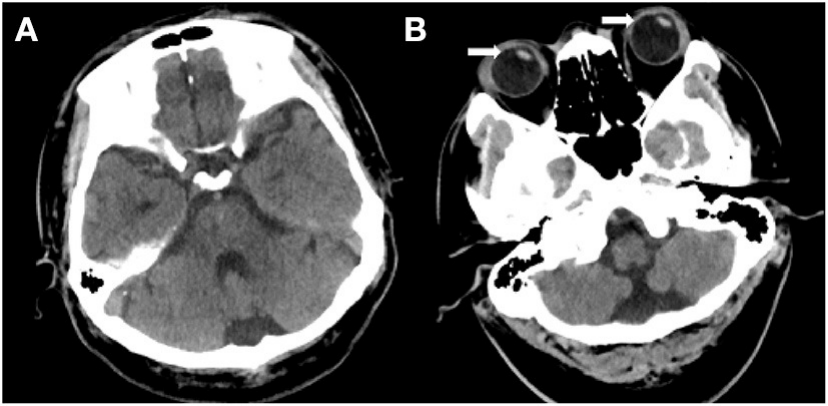
**(A, B)** Cranial computed tomography (CT) demonstrates no abnormality in the brain tissue. **(B)** A leftward conjugate ocular deviation is observed while the eyes are closed (white arrow).

A 42-year-old man presented with acute persistent vertigo, concomitant with nausea, vomiting, and severe gait instability for 6 h prior to admission to the emergency department (ED). He denied experiencing headaches, neck pain, auditory symptoms, diplopia, dysphagia, dysarthria, and other focal neurological symptoms. He had a history of cerebral hemorrhage, hypertension for 7 years, and diabetes mellitus for 5 years. Physical examination revealed spontaneous right-beating horizontal-torsional nystagmus that became more prominent on rightward gaze (Supplementary Video 1). There were no corrective saccades on the bedside head impulse test (HIT), without head tilt or skew deviation (Supplementary Video 2). The patient fell to the left side during the Romberg test. The general physical and remaining neurological examinations yielded normal results. Cranial computed tomography (CT) performed immediately upon arrival to the ED did not provide any (imaging) evidence for ischemia or hemorrhage of the brainstem and cerebellum (Figure 1). However, CT revealed leftward conjugate ocular deviation upon closing the eyes (Figure 1B). Since the bedside HITs were normal, we considered this patient to have a “pseudo-vestibular neuritis” with central HINTS and administered pharmacotherapy for symptom relief.

**Figure 2:**
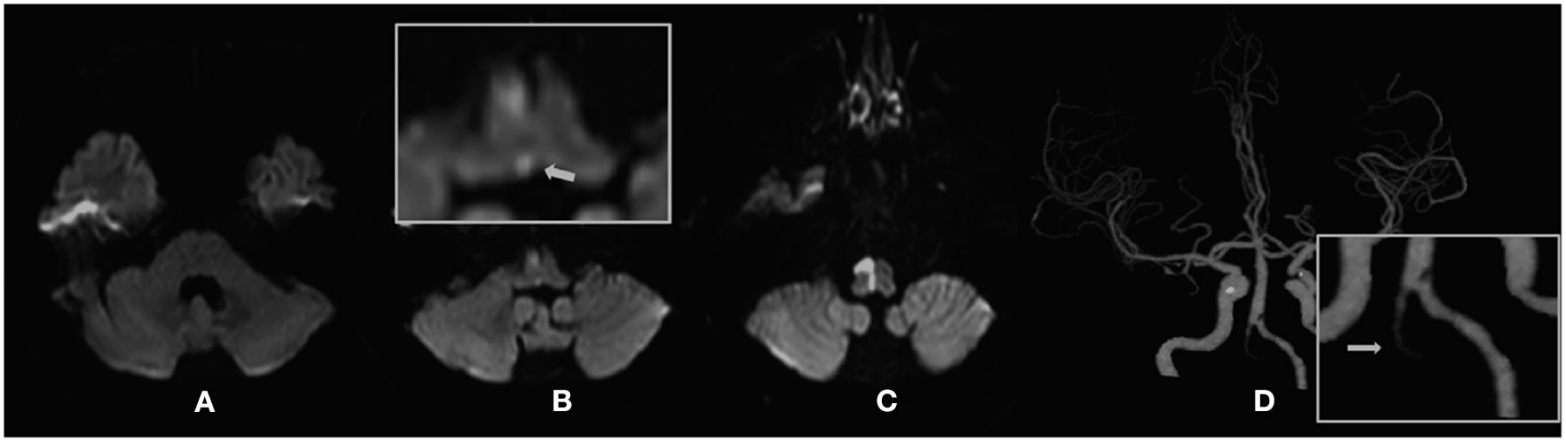
**(A–C)** Diffusion-weighted magnetic resonance imaging (DWI) of the patient. **(A)** No abnormal signal is observed in the vestibular nucleus of the pons. **(B)** Acute infarction in the medial medulla, which involves the nucleus prepositus hypoglossal (NPH) (white arrow). **(D)** Cranial computed tomography angiography (CTA) of the patient demonstrates total occlusion of the left intracranial vertebral artery (ICVA), which represents the offending vessel.

**Figure 3:**
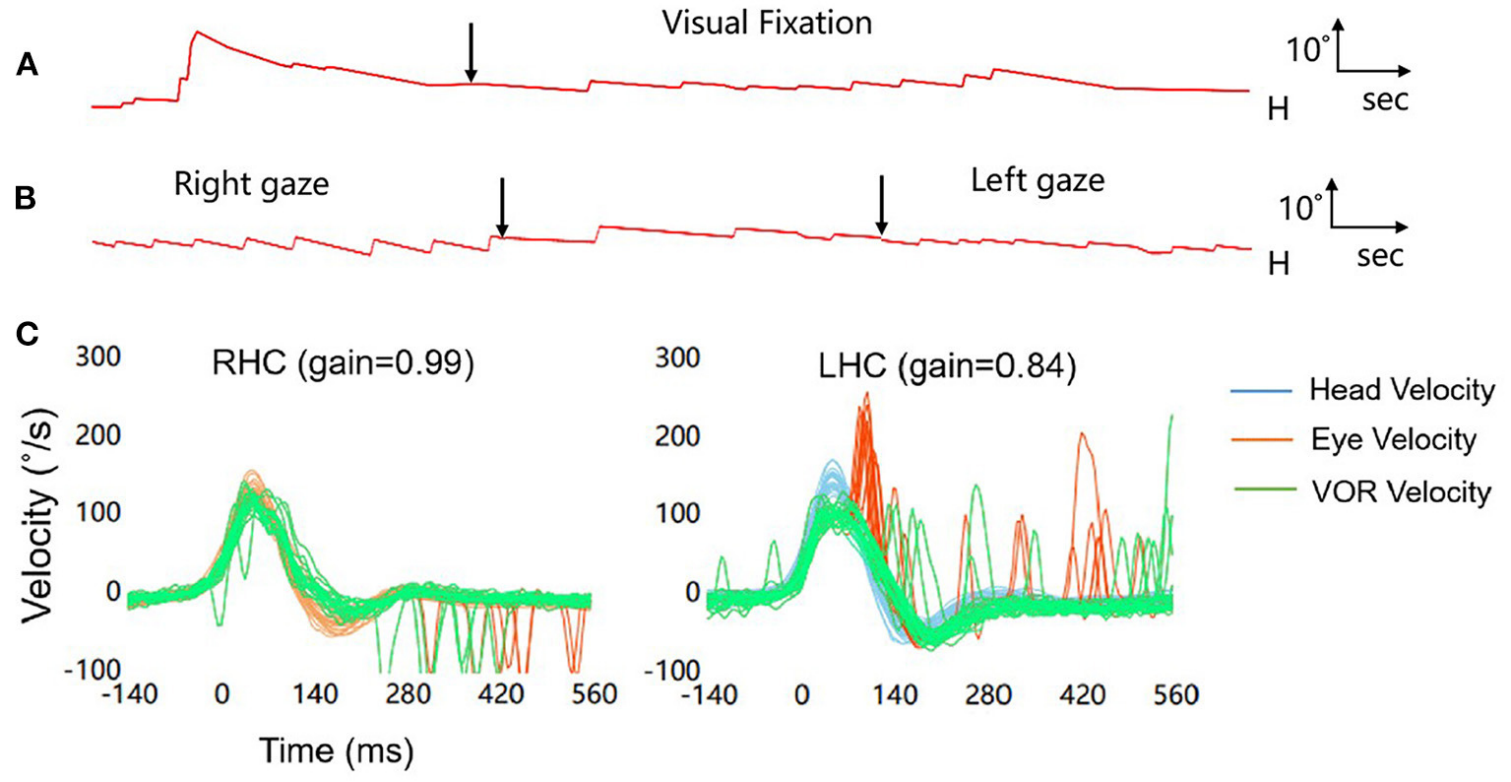
**(A)** Without visual fixation, the patient demonstrates spontaneous right-beating nystagmus with a linear nystagmus waveform and a mean slow-phase velocity (SPV) of 7.7°/s, which decreases during visual fixation, with a mean SPV of 1.6°/s. **(B)** Nystagmus in all gaze directions is similar with spontaneous nystagmus, and is more intense upon staring to the right. **(C)** Results of the video-oculography-based HIT (vHIT) (RHC, right horizontal semicircular canal; LHC, left horizontal semicircular canal). The vHIT results demonstrate numerous corrective saccades in the left horizontal semicircular canal, but the VOR gain is in the normal range, thereby suggesting damage in the central nervous system. vHIT examination of the bilateral anterior semicircular canal and posterior semicircular canal reveals no abnormality.

Four hours following admission, the symptoms underwent exacerbation, manifesting as left-sided limb weakness and dysarthria, without dysphagia. A bedside examination revealed spontaneous right-beating nystagmus, difficulty in extending the tongue to the right, positive left Babinski's sign, and abnormal vibration and position sense in the paralyzed limb; nonetheless, the patient did not have pain and fever. Subsequently, he underwent cranial magnetic resonance imaging (MRI), which revealed an acute infarct confined to the right side of the medial medulla (Figures 2A–C). Cervical and cranial CT angiography revealed occlusion of the left vertebral artery at the V4 segment (Figure 2D). Furthermore, we recorded nystagmus using a video-oculography system (ICS Impulse, Otometrics, Denmark). The patient exhibited spontaneous right-beating nystagmus with a mean slow-phase velocity (SPV) of 7.7°/s, which decreased during visual fixation, with a mean SPV of 1.6°/s (Figure 3A). Nystagmus was identical to spontaneous nystagmus in all gaze directions but was more intense upon gazing to the right (Figure 3B). HIT recording using the ICS Impulse system revealed a normal gain in the vestibulo-ocular reflex (VOR) (0.99 for the right horizontal canal and 0.84 for the left horizontal canal; normal value >0.80), and numerous consistent covert corrective saccades were captured upon turning the head to the left side (Figure 3C). Echocardiography and electrocardiography did not provide any evidence for a cardiogenic etiology.

Following 1 week, the vestibular symptoms and nystagmus had largely disappeared, and the left limb weakness and deep sensory deficits had partially improved; however, he was still unable to walk independently.

## 3. Discussion

The patient presented with central unilateral paralysis of the hypoglossal nerve, contralateral limb hemiparesis, and deep sensory hemianopia; thus, the lesion was localized clinically to the sublingual nucleus, corticospinal tract, and medial tegmental tract. Moreover, cranial MRI revealed an acute cerebral infarct in the medial medulla, which met the diagnostic criteria for Spiller syndrome (1). The initial clinical symptoms and abnormal oculomotor signs presented a diagnostic challenge. This is because infarcts located at this anatomical site present only with symptoms of isolated acute vestibular damage commonly arising from the brainstem root entry zone, vestibular nucleus, or cerebellum (12), but not the medial medulla. Therefore, the functional and anatomical relationship between the signs and symptoms of vertigo and medullary infarction in this patient requires further explanation (12, 13).

**Figure 4:**
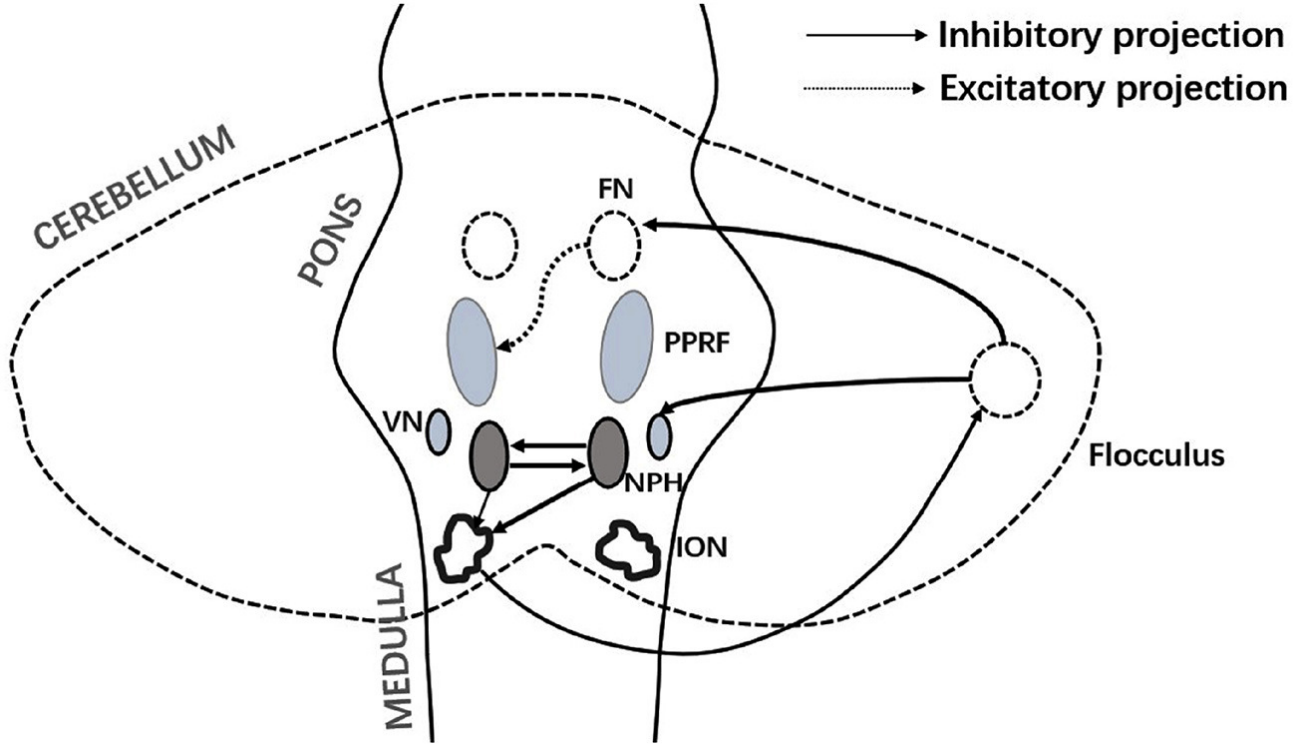
Connections between the NPH and vestibular-brainstem-cerebellar system. FN, fastigial nucleus; VN, vestibular nucleus; NPH, nucleus prepositus hypoglossal; PPRF, paramedian pontine reticular formation; and ION, inferior olivary nucleus. The ION principally accepts inhibitory fibers from the contralateral NPH. Damage to one side of the NPH excites the contralateral Purkinje fibers. The contralateral VN and cerebellar fastigial nucleus are suppressed, and the excitability of the contralateral PPRF increases.

Interestingly, HIT examinations of the patient demonstrated covert left corrective saccades at 4 days following onset. Furthermore, the gain in the VOR was still within the normal range at 4 days, which confirmed the presence of a clear central vestibular compensatory effect (13, 14), thus suggesting the symptoms of vertigo were closely related to the impairment of central vestibular function (11, 15–18). Fibers originating from the nucleus prepositus hypoglossi (NPH) near the dorsal midline of the medulla oblongata can affect contralateral vestibular function by inhibiting the pathway formed by the inferior olive–cerebellar lobule–vestibular nucleus on the contralateral side (12, 19, 20) (Figure 4). Therefore, damage to one side of the NPH leads to vestibular dysfunction on the side opposite to the lesion, vertigo symptoms resembling vestibular neuritis, spontaneous horizontal nystagmus, and vestibular ataxia (12, 20). Moreover, the fibers emanating from the NPH can affect the excitability of the contralateral paramedian pontine reticular formation (PPRF) by affecting the neural pathways between the ipsilateral cerebellar flocculus and fastigial nucleus. Damage to one side of the NPH leads to a decline in the inhibitory effect on the PPRF on the contralateral side, thus increasing excitability; furthermore, the patients may exhibit an ocular contrapulsion (Figure 1B) (21–23). Moreover, an NPH lesion reduces the inhibitory effect on the vestibular nucleus, thus contributing to the establishment of central compensatory function and shortening the duration of the vestibular symptoms.

## 4. Conclusion

This case report described a clinically rare case of progressive classic Spiller syndrome that initially mimicked vestibular neuritis. Clinicians unfamiliar with the NPH may require clarification about the correlation between the initial presentation of contralateral pseudovestibular neuritis to the lesion of this progressive stroke. This case report explains the anatomical site of the NPH and its pathophysiological mechanism in the vestibular-ocular movement pathway such that non-nerve-otology professionals can understand the pathogenesis and clinical manifestations of NPH-mediated “pseudovestibular neuritis.”

## Data availability statement

The original contributions presented in the study are included in the article/Supplementary material, further inquiries can be directed to the corresponding author.

## Ethics statement

Written informed consent was obtained from the individual(s) for the publication of any potentially identifiable images or data included in this article.

## Author contributions

HW and YG mainly wrote this article. TS participated in the revision and provided valuable medical records and professional advice. All authors helped organize the case data and provided valuable advice. All authors contributed to the article and approved the submitted version.
